# Application of Proteomics in Maternal and Neonatal Health: Advancements and Future Directions

**DOI:** 10.1002/prca.70004

**Published:** 2025-03-24

**Authors:** Razan Elkahlout, Sawsan G. A. A. Mohammed, Ahmed Najjar, Thomas Farrell, Hilal Al Rifai, Nader Al‐Dewik, M. Walid Qoronfleh

**Affiliations:** ^1^ Department of Research, Women's Wellness and Research Center Hamad Medical Corporation (HMC) Doha Qatar; ^2^ Translational and Precision Medicine Research, Women's Wellness and Research Center (WWRC) Hamad Medical Corporation (HMC) Doha Qatar; ^3^ QU Health, College of Medicine Qatar University Doha Qatar; ^4^ AtomGen İstanbul Türkiye; ^5^ Healix Lab, Al Khuwair South Muscat Oman; ^6^ Neonatal Intensive Care Unit (NICU), Newborn Screening Unit, Department of Pediatrics and Neonatology, Women's Wellness and Research Center (WWRC) Hamad Medical Corporation (HMC) Doha Qatar; ^7^ Genomics and Precision Medicine (GPM), College of Health & Life Science (CHLS) Hamad Bin Khalifa University (HBKU) Doha Qatar; ^8^ Faculty of Health and Social Care Sciences Kingston University, St. George's University of London London UK; ^9^ Healthcare Research & Policy Division Q3 Research Institute (QRI) Ann Arbor Michigan USA

**Keywords:** AI, biomarker discovery, biomarkers, clinical validation, ethnic populations, maternal health, neonatal health, preterm birth, proteomics, racial disparity

## Abstract

Maternal and neonatal health (women during pregnancy, childbirth, and the postnatal period) presents a spectrum of healthcare challenges, including preterm birth, preeclampsia, intrauterine growth restriction, polycystic ovarian syndrome, and gestational diabetes mellitus. While genomic investigations have shed light on many of these topics, protein biomarker discovery, a pivotal aspect of such research, holds promise in offering insights into disease diagnosis, progression, and prognosis. This review paper aims to explore the landscape of proteomics research pertaining to the aforementioned disorders. In the search for viable biomarkers, existing ones are either outdated or lack specificity and new ones being investigated do not commonly make it to the validation stage. In this review, the reasons for the gap between the biomarker discovery stage and the clinical validation stage are evaluated, in addition to what steps are being taken to mitigate the unexpectedly slow scientific and clinical progress. Notably, this paper also delves into the ethnic disparities found in maternal and neonatal health research, as well as how AI is currently being used to alleviate socioeconomic and ethnic disparities, as well as its advantages for the analysis of large “omics” datasets. We anticipate this investigation will provide critical, invaluable information for researchers, medical professionals, and policy decision‐makers in this field to improve overall maternal and neonatal health outcomes.

## 1 Introduction

Maternal and neonatal health presents several challenges that impact the well‐being of both the mother and the baby. The key concerns in obstetrics include preterm birth (PTB), preeclampsia, intrauterine growth restriction (IUGR), polycystic ovarian syndrome (PCOS), and gestational diabetes mellitus (GDM). Maternal and neonatal mortality rates have largely decreased since 1990, with maternal mortality decreasing by almost 50% between 1990 and 2015 and neonatal mortality decreasing by about 49% between 1990 and 2017 [1]. Despite the large decline in both maternal and neonatal mortality, more than 300,000 women die every year due to reasons related to pregnancy and childbirth, and more than 2.5 million newborns die during the first month of life [2].

Advancements in proteomics technology, specifically high‐throughput systems that analyze proteins, have presented an exciting window of opportunity for the discovery of biomarkers. Biomarkers can be described as biopolymers or biophysical markers that can be measured to identify diseases and/or the pathways leading to disease [3]. One of the key aspects of the current direction of precision medicine translational research is the improvement of disease management through the pursuit of disease‐specific biomarkers [3]. Some of the current obstacles in biomarker discovery include the large gap between copious global research efforts and clinical validation, as well as the advancement of “omics” technologies that produce large, complex datasets that invariably require highly specified knowledge to be analyzed correctly [4]. The aim of this paper is to review recent literature on biomarker discovery in the most pressing disorders in maternal and neonatal health and to evaluate the advancement of potential biomarkers toward clinical validation and commercial use.

The next section will discuss the disorders that are contributing the most to maternal and neonatal mortality and morbidity. Among them are disorders as well as conditions that can overlap and be causative agents for the others. The burden of these pregnancy complications is further exacerbated by a lack of diagnosis, progression and prognosis identifiers, and solutions. Furthermore, the regions most affected by high maternal and neonatal mortality and morbidity rates usually have inadequate access to healthcare and are propagated by expensive and inaccessible treatment options upon diagnosis. In this regard, we firmly believe that the triad of research, collaboration, and policy is paramount to developing evidence‐based practices and equity‐centered strategies to find innovation solutions to achieve maternal and neonatal health goals.

At the heart of maternal and neonatal health concerns is PTB; below is a brief discussion of clinical manifestations and implications of these disorders, followed by proteomics application in these conditions.

## 2 Pregnancy Complications and Their Burden

### 2.1 Preterm Birth

In the year 2020, an estimated 13.4 million babies were born prematurely [5]. For context, in the year 2010, 13.8 million premature births were recorded, meaning that over the course of a decade, the global annual reduction rate of PTB was 0.14% [5]. More than 1 in every 10 newborns is born prematurely, and approximately 1 million newborn deaths occur per year due to PTB [6]. PTB is considered the leading cause of perinatal and neonatal mortality and morbidity, accounting for nearly 75% of perinatal mortality and more than half of long‐term perinatal morbidities [7]. For neonates, PTB is the most common cause of long‐term adverse effects such as neurodevelopmental impairments and respiratory and gastrointestinal disorders [6].

Previously, term birth was defined as delivery between 37 and 42 weeks of gestation; however, following evidence that the neonatal outcomes varied during this 5‐week interval, further classifications have now been implemented [8]. The division includes the following: (1) Term‐birth is considered to be between 39 weeks 0 days and 40 weeks 6 days, (2) Early‐term birth can be defined between 37 weeks 0 days and 38 weeks 6 days, and (3) PTB continues to be a delivery that occurs prior to 37 weeks of gestation [8]. In the case of PTB, the WHO further classifies PTB into two categories based on gestational age, with moderate to late preterm being between 32 and 37 weeks, very preterm from 28 to 31 weeks, and extreme preterm being less than 28 weeks [9].

Common symptoms of PTB are watery vaginal discharge, uterine contractions, menstrual‐like cramps, and lower back pain [10]. It has also been noted that in the case of the cervical length being less than 20 mm before 28 weeks of gestation, PTB is indicated with certainty [10]. Furthermore, if the cervical length is less than 25 mm before 24 weeks of gestation, this could be a strong indicator of PTB [10].

**FIGURE 1:**
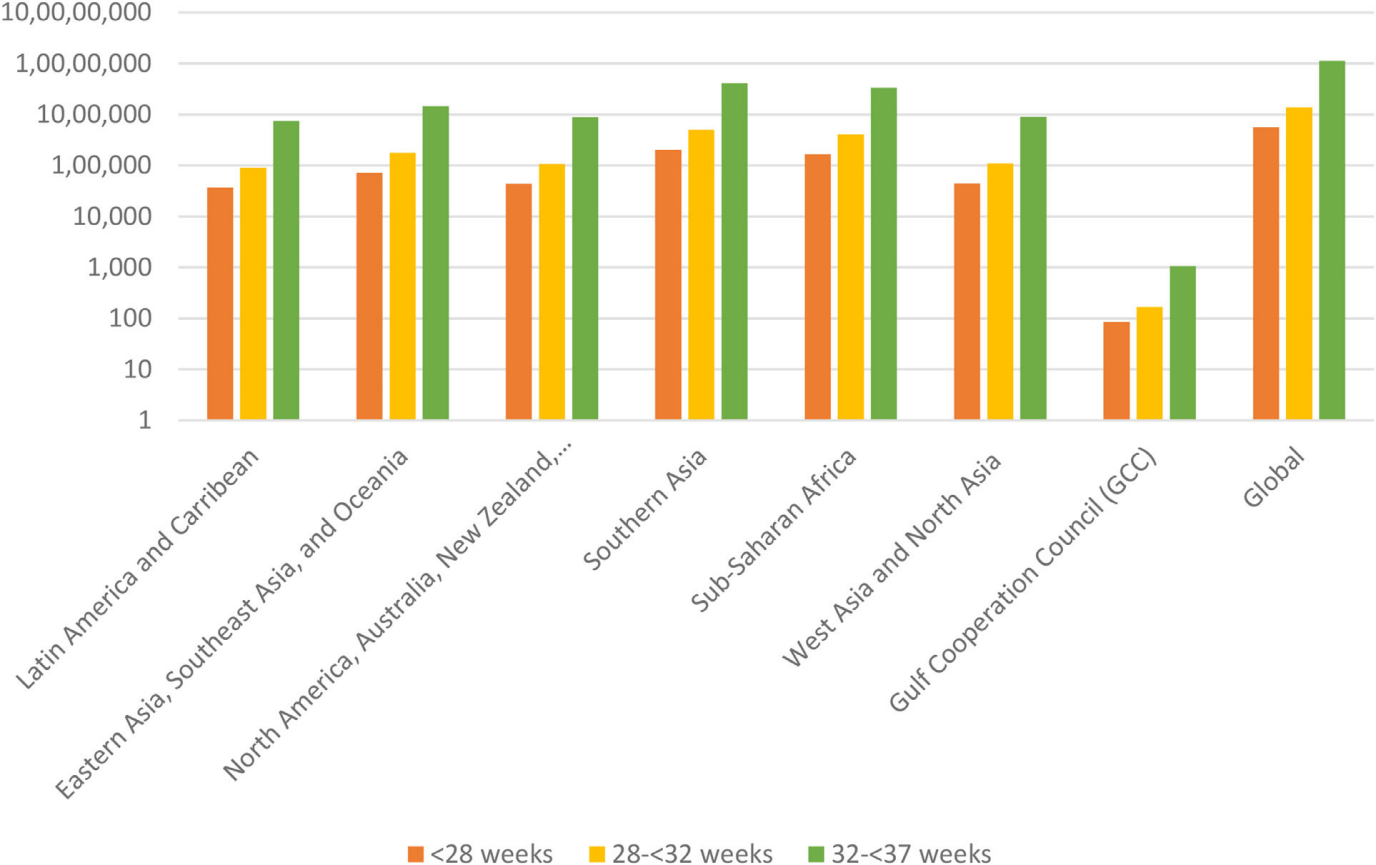
Global and regional incidence of PTB. Graph recreated using a logarithmic scale to showcase data from the supplementary section table “Estimated numbers of subgroups of preterm babies by region” in 2020 by Ohuma et al. [5]. Additionally, GCC statistics are the mean of both Saudi Arabia and Qatar's PTB data [8, 14]. Data from Saudi Arabia ranges from November 2013 to March 2015 in Riyadh. Data from Qatar ranges from April 2017 to March 2018. To note, Qatar's data on the categories <28 weeks and 28 to <32 weeks was unavailable; hence, <28 weeks is only indicative of Saudi Arabian rates of PTB.

In 2020, the global rate of PTB was 9.9%, with just under 15 million babies born prematurely [6]. All but three countries showed an increase in PTB rates between 1990 and 2010 [11]. Leading up to 2020, few countries have decreased their rates of PTB, and those that have seen a marginal reduction of approximately 0.5% per year [5]. Low‐ and middle‐income countries (LMICs) represent a substantial cohort of PTBs in the world, with Southern Asia, sub‐Saharan Africa, and Southeast Asia making up 60% of the total cases [12]. High‐income countries are not far behind. For example, the United States is among the top 10 countries that make up 60% of the global PTB rate and represents sixth highest in rank with approximately 0.5 million PTBs per year [11]. Most recently, the United States’ PTB rate in 2022 was 10.4%, meaning that 1 in every 10 babies was born prematurely [13]. In Qatar, classified as a developing country by the International Monetary Fund, the PTB rate is 8.8%, with the in‐hospital mortality rate being 16.9% [8]. It is worth noting that 85% of PTBs occur between 32 and 37 weeks of gestation, where better outcomes are typically achievable without neonatal intensive care [6]. Global and regional prevalence of PTB is displayed in Figure 1 [5, 8, 14]. In addition to the report from Ohuma et al. [5], we combined Saudi Arabia and Qatar's locally reported rates of PTB in Figure 1 to gain a more accurate picture of where the Gulf Cooperation Council's (GCC) rates of PTB stand in comparison to the global rates.

PTB is thought to have both genetic and environmental factors, and can be influenced by medical conditions, fertility treatments, environmental exposures, and/or behavioral and socioeconomic factors [8]. In the United States, there is a disparity between different racial groups, suggesting that there may be higher risk groups even within countries [13]. Several factors can lead to PTB, which include medically indicated deliveries that are either induced or scheduled via cesarean section (C‐section), spontaneous preterm labor with intact membranes, and preterm premature rupture of membranes (PPROM) [7]. Spontaneous preterm labor is caused by a cohort of pathological processes and notably causes ∼70% of PTBs [15]. The survival rate of preterm neonates is 10% in low‐income countries and 90% in high‐income countries [12]. There is a large disparity between the quality of care, which increases the burden of PTB. PTB is a hallmark complication that can also be linked to several other disorders throughout pregnancy. Disorders such as preeclampsia, IUGR, GDM, and PCOS can all lead to PTB, or may indicate preterm induction as a consequence.

### 2.2 Preeclampsia

Preeclampsia, a hypertensive disorder of pregnancy (HDP), is a complex disease that affects 2%–8% of all pregnancies [16]. In the period of March–December 2017 in Qatar, the incidence of preeclampsia in the State of Qatar was 2.3% [17]. Preeclampsia is marked by hypertension and, more often than not, proteinuria. Preeclampsia can be caused by several factors, including a medical history of certain disorders such as chronic hypertension, social risk factors such as being a part of a minority racial or ethnic group, the means by which the pregnancy came about such as assisted reproduction, and physiological abnormalities [18].

The majority of preeclampsia cases arise at term, and while it may cause mild complications, it usually resolves itself soon after delivery. However, when preeclampsia arises well before term, it can result in life‐threatening or fatal complications. Between 5% and 20% of all preeclampsia cases arise before term [18]. Preeclampsia can lead to several adverse outcomes for both the mother and neonate; it can be uterine and placental dysfunction, which leads to fetal growth restrictions, indicating a low birth weight [16]. What makes preeclampsia exceptionally dangerous are its organ‐damaging effects on the mother, causing conditions like pulmonary edema, hepatic dysfunction, and blood clotting disorders such as thrombocytopenia and disseminated intravascular coagulopathy [16]. IUGR is a condition that can occur as an adverse outcome of preeclampsia and is associated with several other dangerous conditions.

### 2.3 Intrauterine Growth Restriction

IUGR is usually associated with small for gestational age (SGA) condition, which is marked by the birth weight of an infant being below the 10th percentile, and may also include abnormal growth [19]. The overall survival rate of severe and early IUGR (diagnosed before 25 weeks of gestation) is 61.2% [20]. Infants born with IUGR are at a higher risk of developing minor cognitive deficits and metabolic syndromes in future [19]. Cellular energy production and metabolic rate were found to be decreased in the mitochondrial function of IUGR babies [21]. This is a result of low oxygen and nutrient availability, an adaptation that reduces mitochondrial metabolic capacity that is necessary for fetal survival [21]. PTB is linked to these complications and increases the risk of neonatal mortality by two to four times [19]. IUGR affects 5%–10% of all pregnancies [22]. In a month‐long study in 2011 in Qatar, the incidence of IUGR was estimated to be ∼6% [23], which falls within international estimates.

### 2.4 Polycystic Ovary Syndrome

PCOS is an endocrine disorder and is considered one of the leading causes of female infertility. The prevalence of PCOS falls between 4% and 21% depending on the diagnostic criteria, making it the most common disorder among women of reproductive age [24]. With the increased availability of assisted reproductive procedures, women with PCOS are giving birth at almost the same rate of women without [25]. It is a heterogenous genetic disorder; however, the causes remain unknown. Symptoms of PCOS include menstrual and ovarian dysfunction, hyperandrogenism, and metabolic dysfunction [24]. The metabolic dysfunction caused by PCOS can lead to further complications such as type 2 diabetes (T2D), cardiovascular morbidities, and hypertensive disorders [24]. Women with PCOS also experience higher rates of obstetric complications such as obesity, GDM, preeclampsia, and hypertension [24].

PCOS is a relatively underfunded research avenue, though it has been consistently associated with metabolic dysfunction and PTB, and the high morbidity and mortality rates of each. The diagnosis of PCOS can be nonspecific, with the Rotterdam criteria stating that a PCOS diagnosis should include at least two of the following: polycystic ovaries, hyperandrogenism, and the absence of ovulation [25].

### 2.5 Gestational Diabetes Mellitus

GDM is a common, multifactorial disease found in pregnant women, and it is the leading metabolic disease affecting up to 25% of women during pregnancy [26]. GDM is marked by intolerance to glucose that is identified with the onset of pregnancy or is first recognized during pregnancy, and can lead to T2D, as well as impaired pancreatic β‐cell function [27]. Diabetes places women at a higher risk for reproductive dysfunctions, especially when it is also affiliated with conditions such as obesity, PCOS, and hyperinsulinemia [26]. GDM is usually detected late in the second trimester or early during the final trimester [28]. However, the diagnosis of GDM is complex and controversial due to its broad definition, which includes mild impaired glucose tolerance or impaired fasting glucose. With the high prevalence of obesity and diabetes, as well as the social norms changing to a later age for having children, severe hyperglycemia has become a lot more common [28]. Interestingly, in the case of the United States, 29.3% of individuals in the 20–22 age category are pre‐diabetic, and 4.5% have been diagnosed with diabetes [28]. In this context, it is possible that, in the United States, those diagnosed with GDM may actually be representing undiagnosed pre‐pregnancy hyperglycemia [28]. Women in Asia experience an increased risk of GDM because they are ethnically prone to glucose intolerance. This is seen in India, where 5 million cases of GDM arise per year, making it third in rank among the top five countries with high GDM prevalence [29]. In our view, this is suggesting the importance of discovering new ethnic‐specific biomarkers for early detection.

Women with GDM are at a higher risk of HDP and put the fetus at a higher risk of disorders, leading to excessive growth and adiposity [28]. Approximately 5%–10% of women with GDM develop diabetes immediately after pregnancy, and, if not, women who have had GDM are at a much higher risk of developing diabetes in the next 10–20 years than women who never had GDM [30]. Among the risk factors of GDM are pre‐pregnancy body mass index, carbohydrate‐rich diet, age, family history of diabetes mellitus, smoking, alcohol consumption, and so on [29].

**FIGURE 2:**
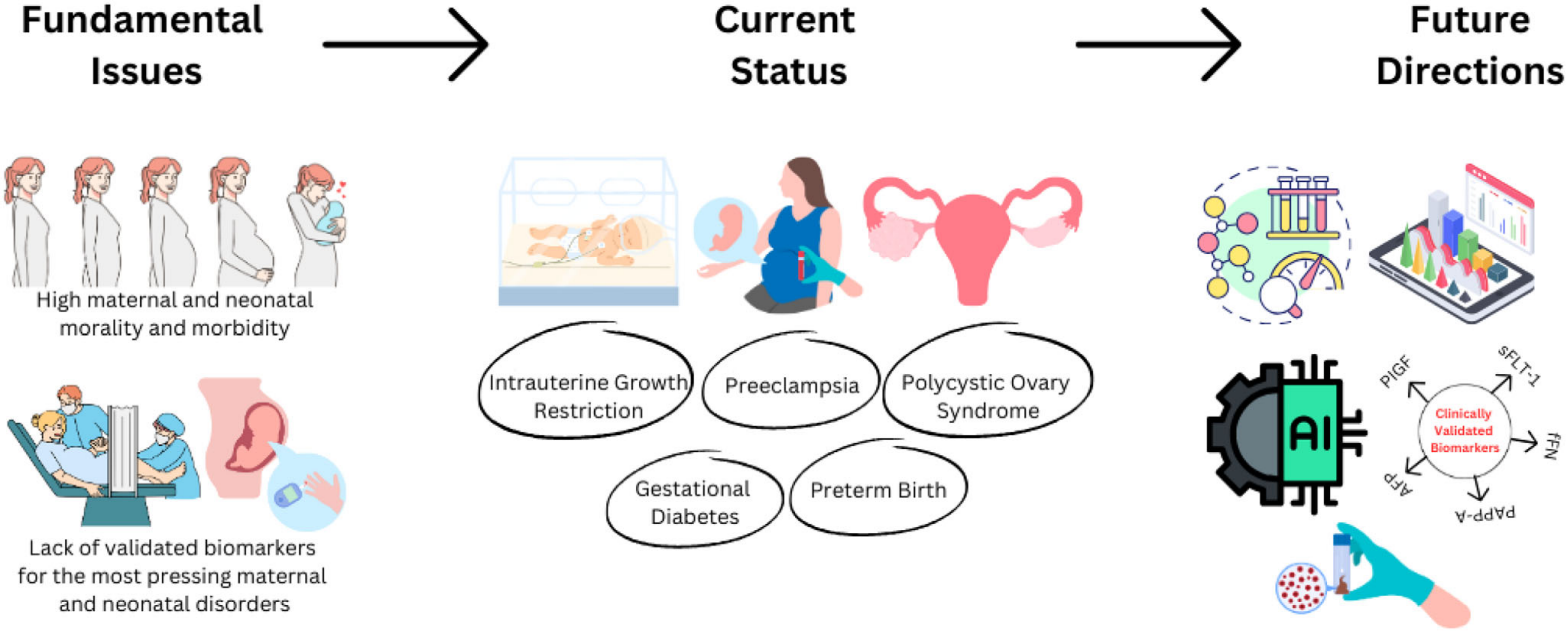
Conceptual framework of key issues and innovations in maternal and neonatal health. This conceptual flowchart highlights and breaks down the current landscape of obstetric biomarker research, the foundational issues, and the largest contributors to high rates of maternal and neonatal mortality and morbidity, as well as potential pathways to mitigate the existing gap between experimental research, clinical validation, and commercial access.

Over the course of this paper, the current research on biomarkers pertaining to each disorder that plays a significant role in the high maternal and neonatal mortality and morbidity rates is discussed. The challenges in the landscape of progress from investigation to clinical validation steps are highlighted. The next section will take a deeper dive into the specific biomarkers related to each disorder, as well as the several challenges such as data analysis of large “omics” datasets, ethnic and economic disparities, as well as the lack of access to healthcare facilities in LMICs. Figure 2 calls attention to the fundamental issues such as the disorders and lack of access to healthcare, the current status of each disorder, specifically in the search for diagnostic and prognostic biomarkers, as well as the future directions that include artificial intelligence (AI), regulatory bodies, further research and a more concerted effort in data analysis to mediate the current standing in this area.

## 3 Proteomics and Its Potential Applications in Maternal and Neonatal Health

**FIGURE 3:**
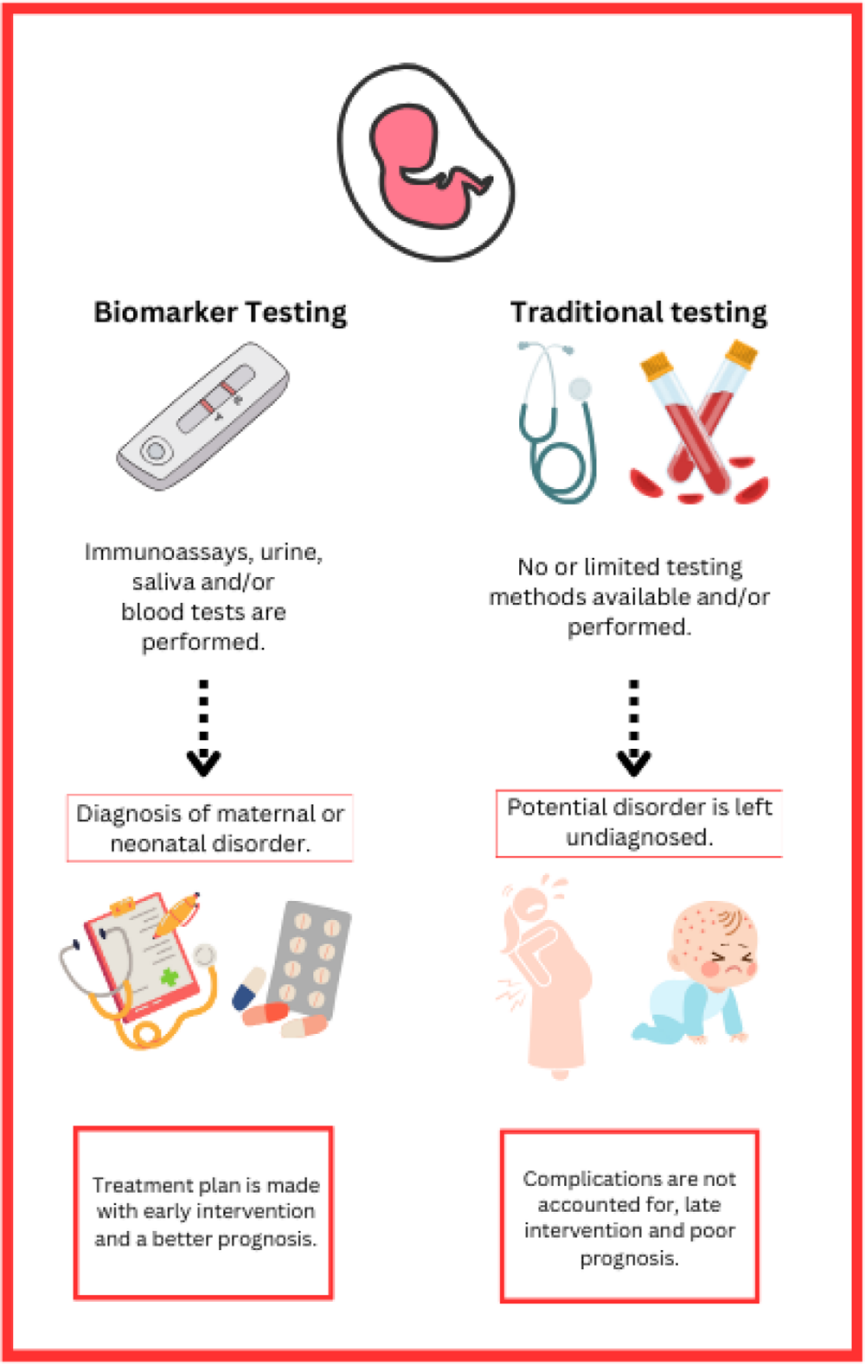
Advantages of biomarker discovery. Biomarker testing is compared to traditional screening methods, showing that biomarker discovery leads to higher rates of diagnosis, which further leads to treatment plans being made early and allowing for a better prognosis. Alternatively, traditional testing can lead to disorders potentially being left undiagnosed or can postpone intervention and lead to a poor prognosis.

Biomarkers are an increasingly effective method of interpreting disease diagnosis, prognosis, and progression [3]. Proteins play a central role in cellular function. They are the primary functional effectors in human biology and are involved in health and diseases, and given that bodily fluids such as serum and urine are sufficient samples, the non‐invasive approach of biomarker discovery provides promising new directions [31]. Understanding biology at the protein level establishes a foundation for the development of diagnostic, prognostic, therapeutic, and preventive medical applications. The advantage of proteomics research is that most diseases, if not all, are likely affected by more than one single gene [32]. However, proteomics allows for the study of the total expressed proteins in cells at any given time. Figure 3 showcases the advantages of biomarker discovery in comparison to traditional testing methods. Notably, the common testing methods and limited biomarkers available in current obstetric healthcare practice lead to a lack of diagnoses and accurate representation of progression and/or prognoses. What truly distinguishes biomarker testing is the notion that a comprehensive treatment plan can be made ahead of time, with complications considered and monitoring occurring throughout the perinatal period. In this paper, we will outline the currently investigated biomarkers involved in PTB, preeclampsia, GDM, IUGR, and PCOS.

One of the promising avenues to consider with biomarker discovery is the potential to screen pregnant women for their susceptibility to such disorders; this should allow for early intervention and better prognosis. Two pregnancy screening approaches are usually contemplated, which include universal screening and selective screening, where either all pregnant women undergo a screening test in the former or the latter being that only women at risk are screened [29]. Currently, common protein‐based prenatal screening includes maternal serum tests for alpha‐fetoprotein (AFP), pregnancy‐associated plasma protein‐A (PAPP‐A), human chorionic gonadotropin, estriol, and inhibin [33]. Additionally, glucose testing is regularly carried out throughout all three trimesters as a monitoring protocol [33].

AFP is a serum protein that is transferred from fetus to mother during the gestational period and can be indicative of several diseases [34]. The concentration of this protein in maternal blood has been linked to genetic conditions such as neural tube defects and Down syndrome [34]. Other disease states have also been implicated in the inhibition of AFP production. Moreover, AFP is also characteristic of the fetal development period and can be used to accurately define the gestational age of the fetus, as its concentration varies throughout the pregnancy period [33]. Interestingly, the common prenatal protein‐based screening tests are limited in their variability for the detection of the most pressing maternal and neonatal health concerns. For example, AFP is mainly used to detect the correct gestational period and to detect Down syndrome and/or other neural tube defects such as spina bifida. Screening tests usually also include PAPP‐A, which is also mostly used to detect trisomy 21 (also known as Down syndrome), as well as trisomy 13 and 18, digynic triploidy, and monosomy X1‐6 [35]. More recently, PAPP‐A has been investigated for its potential use in screening for preeclampsia; however, other maternal serum proteins such as placental growth factor (PlGF) are the preferred biochemical marker [36]. Screening with PAPP‐A in a cohort of approximately 60,000 pregnancies did not improve the prediction of preeclampsia, and in combination with other maternal factors, it actually detected preeclampsia 7.1% less than maternal factors that were paired with PlGF [36]. Multiple marker screenings are not 100% accurate and cannot be used diagnostically in practice. They are universal screening protocols that can allude to whether a pregnancy requires any further testing and/or intervention [33].

Although commercially produced maternal serum biomarker tests exist, they are only regulated by the Clinical Laboratory Improvement Amendments, which have pre‐existing standards that need to be upheld by clinical laboratories developing tests in‐house [37]. The US Food and Drug Administration (FDA) does not regular these tests; therefore, thus far, none of the commercially produced tests are available in the United States. For example, for preeclampsia, there is the Triage PlGF made by Quidel, Elecsys sFlt‑1/PlGF made by Roche Diagnostics, and DELFIA Xpress PlGF 1‐2‐3 made by Perkin Elmer [37]. The Quidel Triage PlGF test and the Elecsys sFlt‐1/PlGF have already been recommended and used in point‐of‐care testing in the National Health Service (NHS) and are approved for laboratory use to diagnose preterm preeclampsia [38]. Commercially produced tests are also available to predict spontaneous PTB such as the PreTRM test by Sera Prognostics, which is conducted during the 19th week of gestation and which uses the maternal serum biomarkers insulin‐like growth factor binding protein‐4 and sex hormone‐binding globulin [37]. The already available screening tests for specific biomarkers such as PAPP‐A and AFP are generally used to predict the onset of several disorders when used in conjunction with different maternal factors; these are available and performed in US hospitals. A functionally similar commercially produced test is the BRAHMS PlGF plus KRYPTOR, produced by ThermoFisher Scientific. This can be used simultaneously with the BRAHMS sFlt‐1 KRYPTOR test to foresee the progression of HDP such as preeclampsia and gestational hypertension, among others [37]. To note, the use of these tests is considered investigational irrespective of the use of algorithmic analysis [37]. Interestingly, the National Institute for Health and Care Excellence in the United Kingdom has recommended that the BRAHMS PlGF and sFlt‐1 KRYPTOR tests not be used routinely in NHS, as high‐quality tests on the accuracy of these tests to predict the onset of preterm preeclampsia are not yet available [38]. The recommended tests for routine clinical use in the NHS are the commercially produced DELFIA Xpress PlGF 1‐2‐3 and the DELFIA Xpress s‐Flt‐1/PlGF 1‐2‐3 ratio assay. Importantly, the DELFIA Xpress tests were previously not recommended for routine use due to a lack of evidence supporting the accuracy of the predictive function of these tests; however, once the large‐scale studies were done, and clear protocols on the intended use of the tests were outlined by the company, the test was approved for use [38]. The serum biomarkers that are quantified in these tests will be discussed in later sections, as they have been and are currently being investigated for their role in several of the disorders outlined in this review.

More recently, Shitara et al. [39] explored the meconium proteome analyzing host‐derived proteins to not only elucidate the gastrointestinal physiology of newborns but also to shed light on the pathophysiology of systemic diseases like gastrointestinal diseases, congenital heart diseases, chromosomal abnormalities, and congenital infection diseases. The study underscores the value of meconium as a non‐invasive clinical sample with significant potential as a biosample for biomarkers discovery for various conditions, particularly PTB. It also explores the relationship between meconium and amniotic fluid proteins, highlighting the potential of amniotic proteomic analysis to predict both maternal and fetal conditions. This study serves as a noteworthy reference in the field.

### 3.1 Biomarker Discovery in PTB

Spontaneous preterm labor and PPROM make up 45% and 25% of all preterm deliveries, respectively [40]. There are many factors that can contribute to spontaneous PTB (sPTB), making it difficult to predict its occurrence during early pregnancy. While a history of PTB is the most well‐known risk factor for reoccurrence, it is not enough to use as a predictive biomarker, although it is used in some parts of the world as the important single factor for screening [40]. Screening using multiple maternal factors that pose a risk to preterm labor, such as advanced maternal age, obesity, smoking, and/or conception through in vitro fertilization, is becoming an increasingly used model when screening for risk of sPTB during the first trimester; however, it has been shown to only predict 20% in nulliparous women and 38% in women with preterm delivery history and/or late pregnancy loss [40].

**FIGURE 4:**
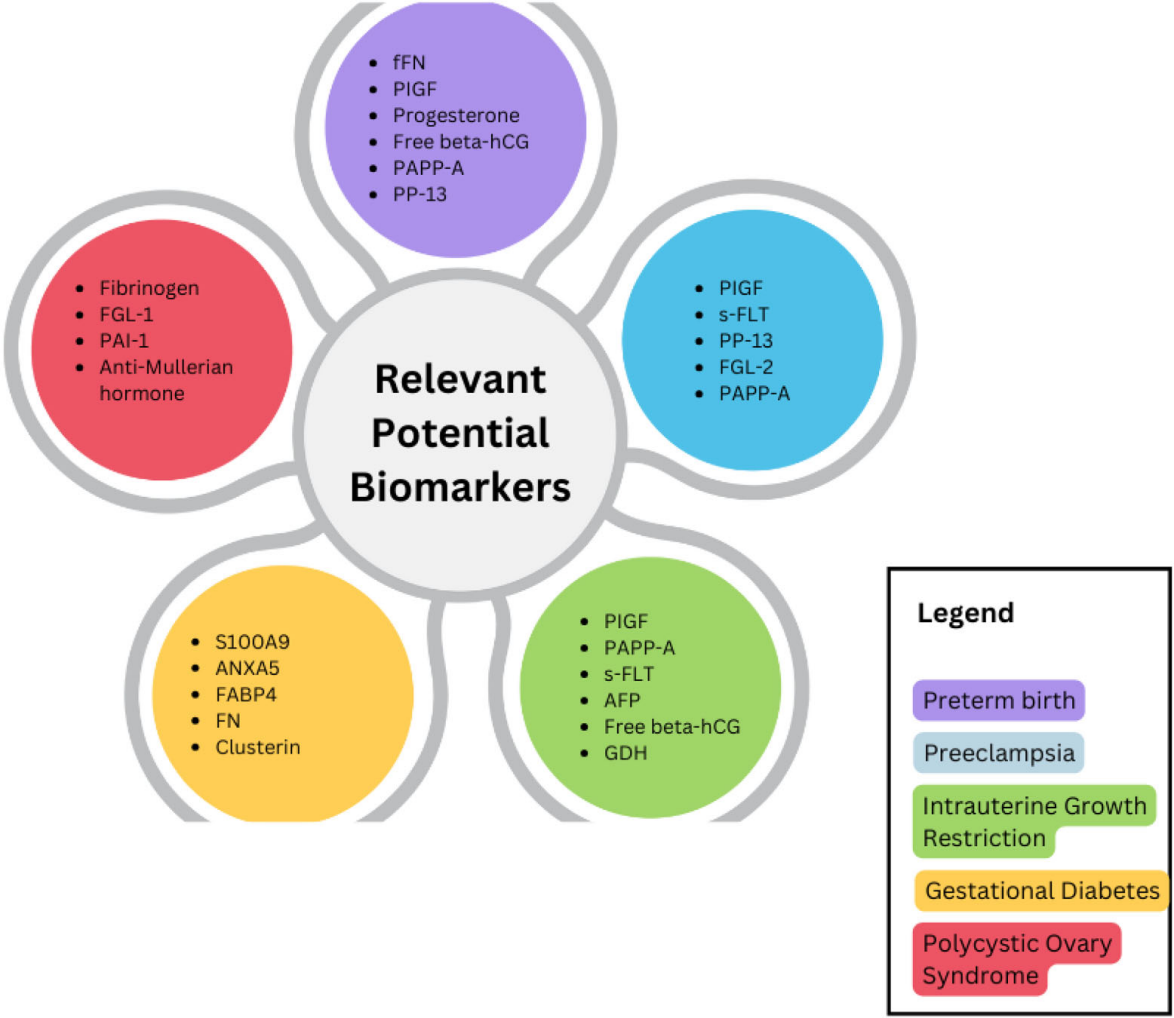
Potentially relevant biomarkers in preterm birth, preeclampsia, intrauterine growth restriction, polycystic ovary syndrome, and gestational diabetes. Biomarkers most recurring in literature searches are listed in line with the disorders they can aid in diagnosing, predicting progress, and evaluating prognosis. To note, some proteins are repeated and can be used to predict the onset of several disorders.

There is evidence that biomarkers that predict preeclampsia may be similar to those that can predict sPTB, as sPTB is suggested to be part of the placental insufficiency spectrum, like preeclampsia [40]. Some proteomic biomarkers may include free β‐human chorionic gonadotropin (free β‐hCG), PAPP‐A, placental growth factor (PlGF), placental protein 13 (PP13), a disintegrin and metalloprotease 12, inhibin‐A, and activin‐A [40]. In the United States, free β‐hCG, PAPP‐A, PlGF, and inhibin are commonly used as part of the prenatal screening tests used as universal biomarkers [33] (Figure 4). PlGF, in combination with several other maternal factors during screening, can be indicative of preeclampsia and henceforth PTB [36]. These serum proteins have been recycled in obstetrics diagnosis protocols over the last decade or so; however, they are mainly used to detect chromosomal abnormalities. Biomarkers specific to the diagnosis, progress, and prognosis of disorders such as PTB are urgently needed to separate and identify the factors that play significant roles in the occurrence of PTB, in contrast to merging all factors involved in other disorders for the prediction of PTB.

Placental alpha‐microglobulin‐1 (PAMG‐1) and fetal fibronectin (fFN) are increasingly used as predictive biomarkers for sPTB. The PAMG‐1 Partosure Test and the Rapid fFN are tests used to predict specifically when a patient may go into labor when they are already suspected to have preterm deliveries due to other complications/existing disorders. In Van Holsbeke et al. [41], tests were used to evaluate their predictive accuracy 7–14 days before delivery. Using these tests, although only predictive in the final stage of pregnancy, they also may help to alleviate unnecessary hospital admissions and drug administrations. In the study by Van Holsbeke et al. [41], the PAMG‐1 test showed a higher accuracy when predicting whether delivery would occur. Both these tests are vaginal biomarkers [42]. Dawes’ et al. [43] study showed that sensitivity was 80% for both qualitative and quantitative fFN tests and only 20% for PAMG‐1 when it came to predict sPTB within the final 7 days before delivery.

Salivary progesterone has been investigated as another biomarker for PTB when Lachelin et al. [44] reported that free progesterone levels were predictive of the risk for PTB. In fact, the American College of Obstetricians and Gynecologists in 2008 suggested progesterone use as a preventative method for PTB [12]. A meta‐analysis performed by Cando et al. [12] collected data from studies done on a total of 861 women from India, Egypt, Iraq, and the United States, where the levels of salivary progesterone were measured after the 28th week of gestation. The results showed with high significance that PTB is marked by lower salivary progesterone levels [12]. Salivary progesterone is a promising biomarker to identify the risk of PTB. Fundamentally, access to affordable and efficient screening exams is at the core of alleviating the burden of PTB on LMICs, which mostly have the highest rates of PTB. Immunoassays that are more accessible may make all the difference, especially in the perspective of the ease of collecting the patient's samples through their saliva, which removes the need for phlebotomists, nurses, and/or the use of valuable equipment to draw serum for example. Notably, the study by Cando et al. [12] requires a much larger cohort to be considered significant; however, the demographic used was also indicative of potentially successful and ethical investigations.

### 3.2 Biomarker Discovery in Preeclampsia

Preeclampsia is a highly heterogeneous disorder of pregnancy, where both maternal and fetal onset can be highly variable, making it difficult to assign a universal biomarker to all cases [45].

Robineau‐Charette et al. [45] have divided preeclampsia into three distinct and clinically relevant subtypes. Cluster 1 includes maternal preeclampsia, where the placenta is healthy, and preeclampsia is driven by both maternal factors and minimal placental dysfunction. Cluster 2 includes canonical preeclampsia and is marked by the current theories underscoring the pathophysiology of preeclampsia, where transcriptional profiles show placental hypoxia, and histological profiles show maternal vascular malperfusion. Cluster 3 includes mainly preeclamptic samples but was marked by transcriptional and histological profiles that included increased immune and pro‐inflammatory genes like tumor necrosis factor‐α and interferon‐γ, as well as placental lesions and maternal‐fetal interface disturbances. Through the transcriptional screening of samples from each cluster, Robineau‐Charette et al. [45] showed that fibrinogen‐like protein‐2 (FGL‐2) was both upregulated and downregulated in distinct cluster groups. FGL‐2 is an immunoregulatory protein and is involved in fibrin deposition. In the context of preeclampsia, the group found that FGL‐2 was downregulated in placentas from Cluster 2 relative to Cluster 1 (which was mainly made up of healthy controls) and upregulated in placentas from Cluster 3 in comparison to Cluster 1 [45].

The FDA has very strict guidelines for the clinical validation of biomarkers, and hence there is a limited number of biomarkers that are qualified to predict or indicate maternal disorders. In 2023, the FDA approved the use of PlGF, PAPP‐A, maternal factors, uterine artery pulsatility index (UtA‐PI), and mean arterial pressure, all in combination, to predict preeclampsia following a study by the Fetal Medicine Foundation (FMF) [46]. The study had a false‐positive rate of 10%; however, all of these factors together were able to successfully indicate 75% of preterm preeclampsia cases and 47% of term preeclampsia [31]. In the first trial, close to 36,000 serum samples were used from pregnant women during 11–13 weeks of gestation. The study was then repeated with approximately 8000 serum samples, where the predictive model was able to predict 100% of early‐onset preeclampsia, 75% of preterm preeclampsia, and 43% of late‐onset preeclampsia. Interestingly, this study also showed that PAPP‐A made no significant differences to the predictive potential of the model [31]. The power of AI analysis may aid in the predictive model performance, where it has fallen short.

In addition to PlGF and PAPP‐A, soluble Fms‐like tyrosine kinase (s‐FLT) and PP13 are the most commonly studied biomarkers in regard to preeclampsia [37, 47]. During normal pregnancies, serum PP‐13 concentration is known to double or triple prior to delivery. In preeclampsia, serum PP‐13 levels significantly decrease, and low concentrations of PP‐13 may be an indicator of the onset of preeclampsia as early as 5–7 weeks [47]. Alternatively, s‐FLT has low sensitivity but high specificity in the accurate prediction of the onset of preeclampsia. This truncated splice variant floats freely in serum, preventing PlGF and vascular endothelial growth factor from binding to their respective transmembrane receptors [48]. The levels of s‐FLT in serum are directly correlated with the severity of preeclampsia and may begin increasing up to 5 weeks prior to the onset of the disease [47] (Figure 4).

### 3.3 Biomarker Discovery in IUGR

IUGR has been shown to affect male and female neonates differently and can lead to perinatal cardiovascular and metabolic complications later in life [49]. Akinyemi et al. [49] compared appropriate for gestational age (AGA) neonates with IUGR neonates and studied the proteomic differences as well as metabolic risk markers to better understand the pathophysiology of this disease and its associated risks. IUGR cord blood proteomes showed differently expressed proteins, specifically in females, mostly implicated with peroxisome pathways. One of the proteins implicated was the catalase enzyme, which in females was slightly increased while remaining unchanged in males. Furthermore, proteins associated with the risk factors such as obesity, T2D, and glucose intolerance were also identified, some of which were unique to females and others unique to males [49].

A murine study done by Pedroso et al. [50] evaluated the effect of IUGR on the protein profiles in the hypothalamus. The results showed that 28 proteins significantly affected hypothalamic proteins and 7 hypothalamic metabolites in adult females that experienced IUGR [50]. The proteins that were downregulated were glutamine synthetase, glutamate carboxylase, glutamate dehydrogenase (GDH), isocitrate dehydrogenase, and alpha‐ketoglutarate. The hypothalamic proteins found to be upregulated in IUGR were NADH dehydrogenase and phosphoenolpyruvate [50]. Most of these proteins are related to mitochondrial function, with many having an analog in humans. As potential biomarkers, a few are discussed further in some detail.

Animal models are commonly used to evaluate the metabolic dysfunction caused by IUGR. These models often mirror the pathophysiological conditions exhibited by human fetuses and serve as a useful tool for proteomics research [21]. In sheep models of IUGR, Pendleton et al. [21] also studied GDH but found its levels to be unchanged in the mitochondria of skeletal muscle. The main difference in these two studies is the focus on the hypothalamus versus the mitochondria in liver and skeletal muscle [21, 50]. They studied a myriad of proteins involved in the mitochondrial processes such as substrate utilization and oxygen phosphorylation on the premise that cellular energy production and the metabolic rate are both decreased in fetuses with IUGR. Interestingly, another study found that immobility stress propagated significant histopathological changes, placental apoptosis, and oxidative stress in murine models [51]. Another study evaluated the use of l‐arginine and *N*‐carbamylglutamate as a supplement to elevate mitochondrial function and reduce duodenal inflammation in IUGR lamb models [52]. The same group also investigated the same supplements for their effect on intestinal function of suckling lambs with IUGR in 2019. The use of these supplements is to target the nitric‐oxide pathway in mitochondrial function, thus decreasing oxidative stress caused by IUGR [53]. Mitochondrial dysfunction is associated with and implicated in the complications caused by IUGR. Future study direction can include monitoring mitochondrial function at different gestational ages through technologies such as the Agilent Seahorse XF Analyzer, specifically the Mito Stress Test, which evaluates mitochondrial function in cells [54]. This is an acceptable standard assay that provides insight into the mechanism of mitochondrial dysfunction.

Prior to the last decade of research, it was thought that maternal serum biomarkers, especially those used for first trimester screening such as PAPP‐A, AFP, and β‐hCG, could be used to identify IUGR. However, recent studies have shown that on their own, as well as in combination, these markers have a low detection rate for SGA [55]. It is evident that many of these biomarkers, along with critical maternal factors, are present and significant in several disorders, and may be used in unison (Figure 4). Lesmes et al. [56] showed a 100%, 76%, and 38% detection rate at <32, 32–36, and ≥37 weeks for SGA neonates. They combined maternal factors, the circumference of both the fetal head and abdomen, femur length, and the serum concentrations of PlGF, sFLT‐1, PAPP‐A, free β‐hCG, and AFP in their screening protocol of 9715 pregnancies, 481 of which were SGA infants. A subsequent study used the same screening protocol, within the same time frame at 19–24 gestational weeks, and added UtA‐PI to the factors evaluated in combination [57]. Albeit small differences, with a total of 7816 pregnancies, 389 of which included SGA neonates, Poon et al. [57] was able to reproduce and improve the detection rates of SGA to 100%, 78%, and 46% during the same time frames as Lesmes et al. [56]. Interestingly, in a meta‐analysis including 432,621 women from 103 studies, the combination of PAPP‐A, free β‐hCG, PlGF, and PP‐13 had a low predictive capacity for SGA [58]. It is evident through the numerous studies that combine different first trimester biomarkers, maternal factors, and fetal biometry that each maternal and neonatal disorder might require a specific screening protocol to diagnose or predict its onset. The different results yielded even within different gestational weeks shed light on the importance of consistent, repeated, and varied research efforts on how minute details could affect the predictive ability of the given biomarkers.

### 3.4 Biomarker Discovery in PCOS

PCOS is associated with several comorbidities and affects 5%–10% of women within the reproductive age. In a study comparing whole protein expression in normal ovaries and ovaries affected by PCOS, it was found that 54 proteins were upregulated in PCOS, while 15 proteins were upregulated in normal ovaries [59]. The proteins that were differentially expressed in PCOS were involved in cellular physiological processes and metabolism, such as homeostasis and proliferation, and the regulation of both cellular processes and metabolism [59]. It is worth noting that several metabolic disorders are linked to PCOS and might present an opportunity for further biomarker discovery that includes shared protein regulation. Hypofibrinolysis and thrombophilia are among the risk factors for spontaneous abortions and complications in pregnancy for PCOS patients, as well as pose an increased risk for cardiovascular diseases in PCOS. Several differentially expressed proteins were implicated in hypofibrinolysis and thrombophilia, which include annexin 2 (A2), a calcium‐dependent phospholipid‐binding protein, antithrombin III, fibrinogen alpha chain, fibrinogen gamma chain, and plasminogen‐related protein A. A2, for example, is downregulated in PCOS and further investigations can provide insight on whether that is associated with oocyte arrest, increased risk of cardiovascular disease, spontaneous abortions, and pregnancy complications [59]. Commonly, PCOS patients have a higher risk for cardiovascular complications, and fibrinogen has been associated with long‐term cardiovascular disease risk [60]. Fibrinogen has been investigated for its association with the persistent health concerns found in patients with PCOS, such as cardiovascular disease and T2D. Ozgokce et al. [60] found that fibrinogen levels were elevated in PCOS patients and were also correlated with insulin resistance. Similarly, a study by Zhang et al. [61] found fibrogen‐like protein 1 (FGL‐1) to be generally associated with PCOS and suggested its elevated levels to be considered a clinical biomarker for PCOS. Moreover, they also found further correlation between FGL‐1 and insulin resistance, T2D, and obesity (Figure 4).

Interestingly, further studies have also been conducted on the plasminogen system and its association with PCOS using animal models. In a study by Ma et al. [59], plasminogen‐related protein A was upregulated in PCOS and associated with hypofibrinloysis and thrombosis. In a murine study, there was a higher concentration of plasminogen activator inhibitor‐1 (PAI‐1) in rats with letrozole‐induced PCOS [62]. Following letrozole induction, the rats were then treated with the angiotensin‐converting enzyme inhibitor, lisinopril, which in turn resulted in a lower concentration of PAI‐1, which is hypothesized to be because lisinopril inhibits the systemic and ovarian production of PAI‐1 [62]. In another murine study, the plasminogen system was monitored throughout the estrous cycle in hyperandrogenized mice. Notably, PAI‐1 expression was fluid throughout the beginning of the cycle, increasing at the same time and rate as the control group, thought to be a necessary step; however, as the cycle progresses and the control group begins to taper the expression of PAI‐1, its concentration continues to increase in PCOS model mice, supporting PAI‐1's implication in PCOS [63] (Figure 4).

PCOS is also thought to be associated with hyperinsulinemia, with studies increasingly showing patients with insulin resistance, abnormal glucose metabolism, and T2D. In the same study, Ma et al. [59] found several proteins that are associated with insulin regulation and function that are differentially expressed in normal ovaries versus PCOS ovaries. The proteins mentioned in the study are as follows: FUSE binding protein 1, flotillin‐1, glyoxylate reductase, malate dehydrogenase, and methionine adenosyltransferase II. While there is no direct link between methionine adenosyltransferase II and hyperinsulinemia, previous studies have shown it be involved in hyper‐homocysteinemia, which is a well‐known risk factor for patients with T2D. Methionine adenosyltransferase was downregulated in the ovaries of patients with PCOS [59].

In a study by Gunn et al. [64], 40 adolescent PCOS patients were routinely checked throughout the course of the study, with baseline measurements and subsequent annual checkups that included oral glucose tolerance, psychological, pubertal, and anthropometric tests. Furthermore, urine samples were taken for discovery proteomic profiling. The results showed that two‐thirds of the patients had increased expression of anti‐Müllerian hormone (Figure 4), while three‐quarters showed an increased free androgen index [64]. Interestingly, the inflammatory markers, C‐reactive protein and erythrocyte sedimentation rate, showed increased inflammation in 40% of the patients [64].

### 3.5 Biomarker Discovery in GDM

In GDM, the proteome seems to be altered, where certain proteins are either up‐ or downregulated, or several proteins can be differentially expressed, especially in relation to glucose and lipid metabolism, inflammation, and oxidative stress [29]. The traditional screening method for GDM occurs during the second and third trimester of pregnancy and is done through an oral glucose tolerance test (OGTT) test. The OGTT tests presents limitations as it requires three blood samples before a diagnosis is made, and different organizations such as the WHO and the International Association of the Diabetes and Pregnancy Study Groups have altered the cutoff points over the years, highlighting a difficulty to diagnose and identify GDM [65].

One particular study investigated the proteome in maternal omental adipose tissue. Oliva et al. [66] used 2D‐difference gel electrophoresis to identify novel proteins in maternal adipose tissue that are associated with GDM. They found four differentially expressed proteins that were upregulated by 1.4‐fold and had a *p* value that was less than 0.05. These were significantly higher in adipose tissue obtained from GDM patients [66]. The four proteins were collagen alpha‐2(VI) chain (CO6A2(COL6A2)), fibrinogen beta chain (FIBB), Lumican (LUM), and S100 calcium binding protein A9 (S100A9) [66]. Following this study, no follow‐up investigations on collagen alpha‐2(VI) chain (CO6A2(COL6A2), LUM, or FIBB occurred. However, in a study by Ramachandrarao et al. [67], S100A9 was significantly increased in both GDM and pre‐gestational T2D, in comparison to healthy control samples at 20 weeks’ gestation. Furthermore, in the same study, S100A9 protein peptide counts were independently correlated with maternal obesity [67].

Ten proteins were also found to be significantly lower in adipose tissue collected from GDM patients. These were alpha‐1‐anti‐trypsin (AIAT (SERPINA1)), annexin A5 (ANXA5), fatty acid‐binding protein (FABP4), adipocyte (FABP4), glutathione S‐transferase P (GSTP (GSTP1)), heat‐shock protein 27 (HSP27 (HSPB1)), lactate dehydrogenase B chain (LDHB), peilipin‐1 (PLIN1), peroxiredoxin‐6 (PRX6), selenium‐binding protein 1 (SBP1), and vinculin (VINC) [66]. ANXA5 has more recently been assessed for its role in fetal and placental vascular thrombosis in GDM. The expression of ANXA5 was also significantly downregulated in GDM patients in comparison to healthy controls, suggesting a role in the etiology of the disorder and a need for further investigation as a potential biomarker [68]. Interestingly, several studies have implicated FABP4 in GDM; however, in contrast to the study by Oliva et al. [66], more recent investigations have shown a correlation between elevated levels of FABP4 and the development of GDM [69, 70]. Given the involvement of FABP4 in lipid pathways and insulin resistance, further studies on how the expression of FABP4 is changed throughout pregnancy can shed light on the development of GDM. In the study by Duan et al. [70], the group used the adipocyte FABP inhibitor to assess whether symptoms of GDM would decrease. Similar treatment protocols can be used in the event of further investigation and clinical validation of biomarkers such as FABP4 (Figure 4).

A study by Nagalla et al. [30] showed promising results with two potential biomarkers identified. Potential changes in glycosylation of the glycoprotein's fibronectin and pregnancy‐specific glycoprotein‐1 (PSG‐1) were analyzed in maternal serum. Fibronectin glycosylation with *Sambucus nigra* lectin (SNA) binding was found to be an efficient early predictor of GDM. Ai et al. [65] was able to isolate the protein biomarker Clusterin, which showed a significant change in patients with GDM, in addition to functionally protecting cells from oxidative stress in pregnancy and playing a crucial role in lipid metabolism, highlighting its relation to the development of GDM.

## 4 The Challenge of Clinical Validation

The pathway from biomarker discovery to clinical validation is complicated by a myriad of factors. While the list of candidate biomarkers is long, only few make it to the final stages. The nature and functionality of biomarkers are diverse, ranging from molecular, histologic, pathologic, and imaging, while also playing roles in diagnosis, prognosis, and disease progression [71]. While biomarkers are potentially transformative and powerful tools for disease diagnosis and treatment, further and specific validation is needed before the premature assumption of them being indicative of real clinical outcomes. The biomarker clinical utilization pathway has been reviewed recently [3]. In this paper, discussing the relevance of biomarkers to healthcare and precision medicine, their sixth figure exhibits the biomarker development pipeline path and the different relationships and factors that are involved for uptake in clinical medicine.

In a widely known and notable case, the FDA approved the diabetes drug rosiglitazone (Avandia) with the use of hemoglobin A1C (Hb1Ac) as a surrogate biomarker. In trials, the drug was able to reduce the aggregation of Hb1AC. While this was a clinically sound assumption, there was no clearly defined outcome to the reduction of blood sugar in relation to kidney damage, heart attacks, and/or blindness. Rosiglitazone, widely used, was later found to increase the risk of cardiovascular diseases [71].

There are many challenges when it comes to bridging the gap between research and clinical use. As the omics high‐throughput technologies have advanced while simultaneously becoming more accessible, the window of research opportunity has widened. However, with that advancement has come complex datasets that require purely statistics and expert knowledge‐based approaches, making it challenging to extract meaningful molecular signatures [4].

In the case of salivary progesterone as a potential biomarker for PTB in asymptomatic women, saliva samples are readily available and commonly collected as per normal practice [72]. In the search for biomarkers, implementation in clinical practice should be a high priority, making the test more accessible to the lower‐income countries that are most affected by sPTB and that produce the highest mortality rates. Early prediction can lead to prevention, as well as improve the management of clinical movement to an appropriate tertiary referral center in the case of impending labor [72].

## 5 Biomarker Discovery in Ethnic Populations

The most prominent racial and ethnic disparities in healthcare exist in the United States, where both Black and Hispanic women are more likely to be disadvantaged in their access to healthcare, and in maternal and neonatal morbidity and mortality outcomes [73, 74]. Interestingly, in contrast to most obstetric health concerns, in the case of racial disparity, maternal mortality has increased over time [75]. Black women in the United States experience higher rates of maternal and neonatal morbidity and mortality, fetal demise, PTB, low birthweight (LBW), C‐section delivery, hypertensive disorders, and fetal growth restriction [73]. For example, Black women, in comparison to their White counterparts, are twice as likely to deliver preterm infants and/or deliver with LBW [73]. Another example of healthcare inequality is the Arab/Middle East and North Africa community in the United States, which is well studied and documented [76, 77, 78]. While many elements may play a role in racial disparities that effect access to healthcare, such as socioeconomic factors, health behaviors, geographic location, and medical risk factors, these do not represent all the components that affect these outcomes [73, 79]. Ethnic disparity exists not only in Northern America but also in the GCC, but these equity studies are either scarce, very old, or even biased, with most focusing on the healthcare system [80, 81, 82, 83]. Ethnic healthcare concerns have been voiced regarding GCC nationals versus its expatriate members as well as the migrant worker community. Interestingly, a study by Goodman [84], on the development of the healthcare system in Qatar, showed that racial disparities may also exist between the Qatari nationals and the expatriate community. Of the 21 neonatal mortality cases between 2008 and 2011 in the Women's Hospital in Qatar, 15 involved non‐Qatari nationals [84].

In a study by Janevic et al. [85], data from infants born between January 2010 and December 2014 were collected from approximately 500,000 preterm neonates. The aim of the study was to assess the ethnic disparity in neonatal morbidity and mortality, and to investigate the rates of disease among Hispanic, Asian, and Black infants in comparison to White neonates. In specific, they studied the prevalence of necrotizing enterocolitis, intraventricular hemorrhage, bronchopulmonary dysplasia, and retinopathy of prematurity. In all four comorbidities, infants from ethnic backgrounds had an increased risk of developing it, suggesting that the racial and ethnic disparities in neonatal morbidities and mortalities are larger than previously thought [85].

Medical research and education has created an environment in which healthcare and the pharmaceutical industry are oftentimes shaped by a patient's skin color and ethnicity [86]. Biomarkers are an increasingly used avenue in both biological and social science research, with the social sciences using them to track and forecast health outcomes across the population [86]. With the introduction of omics data to biomarker discovery and research efforts over the last few decades, this has propelled social science research to make connections between biological processes and the disparities in health between different groups of people [86].

In the design of research studies, disease etiology and aims of a study need to include and identify racial differences that may be present. The emergence of ethnic in life sciences research has somewhat revolutionized omics research potential and has created a vast movement toward training models with large datasets to utilize the technology and analysis capabilities within subsets of AI like machine learning (ML) and deep learning (DL) [87]. While several obstacles exist in the use of AI such as data availability, quality, and bias, the ethical considerations are a pressing issue for global regulatory authorities [87]. For example, the FDA in the United States released an action plan named “AI/ML Software‐as‐a‐Medical‐Device,” which aims to regulate and requires the relevant national teams to release the datasets they use to train AI and ML algorithms [87]. Notably, this includes a call for detailed breakdowns of the demographics included in these datasets, which also places pressure on researchers to diversify their datasets and disclose their methodology [87]. With the emergence of new technologies like AI, ML, and DL and their use in medical research, there is always going to be public scrutiny and distrust. Regulatory authorities taking ethical concerns seriously will allow for the trust of the public in the use of these technologies, while also bridging the gap that exists in ethnic and racial consideration. Big data, with the assistance of ML and DL, can be propelled to exceed the limitations of current data analysis approaches and to factor in variables such as ethnicity and race to improve translational and precision medicine. Currently, AI is still an emerging technology in the discovery of biomarkers, and the only AI algorithms that have been cleared by the FDA are for breast and lung cancer research [87]. Pending the availability of more data and with a wide demographic range, AI‐driven biomarker research may play the most pivotal role in decreasing ethnic and racial disparities in biomedical research and, by proxy, healthcare services and awareness.

## 6 Using AI for Biomarker Discovery

AI is the hallmark of the 21^st^ century, designed to mimic intelligent behavior by creating computer programs that perform a range of tasks [88]. There is an immense potential for the use of AI to improve healthcare through the tailored analysis of omics datasets and individualized patient information [89, 90].

AI can be classified into two categories: capability‐based AI and functionality‐based AI [88]. Artificial neural networks (ANNs) are the most commonly used model in AI for data classification and analysis. ANNs can be feedforward artificial networks, recurrent neural networks, long‐ or short‐term memory neural networks, convolutional neural networks, and generative adversarial networks [88]. In the scope of biomarker discovery, the different ANNs can be used to “remember” data, break it down into layers, and/or prove or disprove data based on ML [88]. The interpretation of large omics datasets by ANNs creates the prospective of higher standard, accurate, and individualized analysis [91]. Relatively recently, only two novel markers made it past the discovery stage and received FDA approval every year [92]. To bridge the gap between biomarker discovery and clinical validation, there is an urgent call for more data analysis, while a discrepancy exists in the expertise and accuracy needed for such large datasets. AI can be a transformative tool in this endeavor, alleviating a major setback in the progress of clinically validating biomarkers.

Other than closing the gap between research and validation of biomarkers, AI can also be a useful tool in addressing the disparities in the rate of maternal and neonatal morbidities and mortalities among LMICs and high‐income countries. Neonatal morbidity is nine times higher in LMICs than in high‐income countries due to iniquitous access to proper healthcare both during pregnancy and after [93]. Earlier in this paper, we discussed the potential use of AI in bridging the gap between ethnic disparities that exist within countries. LMICs such as countries in Africa, as well as Pakistan, India, and Afghanistan, have already started to investigate how AI and DL can be utilized in using existing data from avenues such as electronic health records and digital health applications to improve maternal and neonatal care [93]. Notably, studies have already begun in the use of neural networks for the prediction of pregnancy and health risk [94]. Raza et al. [94] produced a novel deep neural network architecture that would predict maternal health concerns using health data records. They used 1218 samples from hospitals and community clinics, while also accounting for minorities within the samples through the use of a synthetic minority oversampling technique and aimed to provide results with 98% accuracy [94]. The impact of this study comes from its deep investigation into the performance of a myriad of models and combines them to create an effective ML models’ training [94].

Studies have also already been underway in the prediction of specific maternal disorders such as preeclampsia. A study conducted in Indonesia aimed to develop a prognostic prediction model using publicly accessed health data records with over 3000 patient records [95]. Of importance, the group was able to validate the DL model they used in the prediction of early‐onset preeclampsia while comparing their results to six different prediction models that had already been reported on [95]. The benefit of using AI, and specifically DL models, is the attention to sensitivity, precision, and specificity in obtaining predictive models while also maintaining speed and efficiency in the rate at which research is occurring in this avenue. The prospective use of AI is also not limited to prognostic potential and has also been utilized to find links between different disorders, predict the need for treatment and/or effectiveness of treatment, and define the progression of disease. One study was able to forecast the requirement of insulin treatment in GDM based on information such as body mass index and the results of oral glucose tolerance test (OGTT), and it was able to ascertain that 15%–30% of women with GDM would require insulin treatment using an ML‐based model [96].

## 7 Maternal and Neonatal Health Policy Perspective

As a final thought, it is rare in a scientific or technical article to offer or allude to a policy framework. However, most of the maternal and neonatal health burden resides in LMICs. A program that promotes research and policy/advocacy collaborations that involves multi‐sectoral stakeholders is indispensable for better healthcare outcomes. The strategies in place and the work being carried out, for instance, by the agencies such as USAID [97], UNICEF [2], and the Gates Foundation [98] are precious. The interventions are yielding positive results and making a significant impact on lives.

## 8 Conclusion

The topics covered within this paper are vast yet vital. Research on maternal and neonatal healthcare has been pertinent for decades and has assisted in decreasing the global maternal and neonatal mortality and morbidity rates globally. However, the rate at which this decrease is occurring is relatively slow considering the extensive knowledge, technology, and information available. In specific, biomarkers are among the most frequently used healthcare practices for all diseases, disorders, and conditions, but their use in obstetric health is very limited and requires further validation. More importantly, they need to be made accessible to LMICs that make up most of the maternal and neonatal morbidities and mortalities.

What makes the process of developing and validating biomarkers difficult in the case of the disorders discussed in this paper is that there are numerous, complex links that can be made among them and, in turn, with other diseases. Moreover, there are several causative agents, and the pathophysiology of these disorders might need to be tackled and identified either with unique biomarkers or through the combined use of multiple biomarkers for accurate results. The advantage of using proteomics to fast‐track biomarker discovery is to find the associations between the different disorders or the different risk factors. In addition to omics data that can be produced, AI can also be combined with current research efforts to optimize data analysis and create room for highly accurate, specific, and sensitive results. Importantly, the use of AI has also made room for ethical considerations about ethnic and socioeconomic disparities in the literature, with regulatory authorities requiring demographic data, which will encourage inclusivity.

## Author Contributions

R.E. performed initial research, collected information, and generated short write‐ups. S.G.A.A.M., A.N., T.F., and H.A.R. provided research insight, examined the content, and supported in numerous aspects during the manuscript development process. M.W.Q. and N.A.D. contributed to conceptual work, framework, final draft write‐up, critical reading, and editing. All authors read and approved the final manuscript.

## Ethics Statement

The authors have nothing to report.

## Consent

The authors have nothing to report.

## Conflicts of Interest

The authors declare no conflicts of interest.

## Data Availability

The authors have nothing to report.
